# Construction of a prognostic value model in papillary renal cell carcinoma by immune-related genes

**DOI:** 10.1097/MD.0000000000024903

**Published:** 2021-03-26

**Authors:** Leilei Wang, Weile Gu, Huijun Ni

**Affiliations:** a Department of Laboratory Medicine; b Blood Transfusion Department; c Department of Pharmacy, Traditional Chinese Medical Hospital of Huangdao District, Qingdao, P.R. China.

**Keywords:** immune-related genes (IRGs), papillary renal cell carcinoma (PRCC), prognosis

## Abstract

Papillary renal cell carcinoma (PRCC) is the second most common type of renal carcinoma following clear cell renal cell carcinoma, and the role of immune-related genes (IRGs) in tumorigenesis and metastasis is evident; its prognostic value in PRCC remains unclear. In this study, we downloaded the gene expression profiles and clinical data of patients with PRCC from The Cancer Genome Atlas (TCGA) database and obtained IRGs from the ImmPort database. A total of 371 differentially expressed IRGs (DEIRGs) were discovered between PRCC and normal kidney tissues. Prognostic DEIRGs (PDEIRGs) were identified by univariate Cox regression analysis. Then, we screened the four most representative PDEIRGs (IL13RA2, CCL19, BIRC5, and INHBE) and used them to construct a risk model to predict the prognosis of patients with PRCC. This model precisely stratified survival outcome and accurately identified mutation burden in PRCC. Thus, our results suggest that these four PDEIRGs are available prognostic predictors for PRCC. They could be used to assess the prognosis and to guide individualized treatments for patients with PRCC.

## 1 Introduction

Renal cell carcinoma (RCC) is characterized by a lack of early warning signs (which results in a high proportion of patients with metastases), diverse clinical manifestations, and resistance to radiotherapy and chemotherapy, and there is a potential role for immunomodulation in the inhibition of tumor growth.^[1,2]^ Papillary renal cell carcinoma (PRCC), accounting for ∼15% of kidney cancers, is the second most common type of renal carcinoma following clear cell renal cell carcinoma.^[3]^ Although comprehensive treatments for PRCC have improved, the overall survival rate (OSR) is still low, especially for advanced or metastatic patients for whom treatment options unfortunately remain limited.^[4,5]^ Hence, it is necessary to recognize biomarkers and improve the prognosis of patients with PRCC.

With the development of microarray and sequencing technology, as well as available open-access databases such as The Cancer Genome Atlas (TCGA) and Gene Expression Omnibus (GEO), the discovery of biomarkers and identification of molecular subtypes have been implemented in several cancers.^[6,7]^ There is growing evidence that the immune system plays an important role in the development and progression of cancer.^[8,9]^ The immune system has been reported to participate in different stages of cancer, and some immune checkpoint molecules (such as PD-1, PD-L1, and CTLA-4) are practicable targets of immunotherapy.^[10,11]^ In addition, immune escape has also been confirmed to be a mechanistic marker for chemotherapy resistance in cancer.^[12]^ Immune checkpoint inhibitors reduced the immune escape of cancer cells and enhanced the tumor-specific immune response to attenuate tumor growth.^[13,14]^ Therefore, the identification of relevant IRGs might reduce drug resistance and extend survival time for PRCC.

Recently, researchers have identified numerous IRGs of patients with RCC based on gene expression profiles and have constructed prognostic multigene signatures that can divide patients into different risk groups.^[15–17]^ Previous studies have shown that IRGs were associated with the immune response intensity and predicted the prognosis of patients with PRCC.^[18,19]^ Zhang et al reported that IDO1 and PD-L2 were associated with a poor prognosis in PRCC.^[20]^ However, the molecular events of IRGs in PRCC need to be further explored and summarized, which will lead to the discovery of their potential functions in patients with PRCC.

In this study, TCGA, ImmPort databases and the clinical features of patients were analyzed to construct a prognostic prediction model for PRCC based on IRGs. This could be used to assist clinical treatment by evaluating the prognosis and providing therapeutic strategies for patients with refractory PRCC.

## 2 Materials and methods

Ethics committee approval was not required because all clinical data in this study were obtained from a public database and are available without individual identity.

### 2.1 Data collection and processing

The transcriptomic data, mutation data, clinical and follow-up information from 289 patients with PRCC and the transcriptomic data from 32 normal patients were downloaded from the TCGA portal (https://portal.gdc.cancer.gov/). The IRGs were obtained from the ImmPort database (https://www.immport.org/home). Immune infiltrate data were obtained from the Cistrome project (http://www.cistrome.org/), which contains the abundances of six tumor-infiltrating immune cells (B cells, CD4+ T cells, CD8+ T cells, neutrophils, macrophages and dendritic cells) in PRCC. All of the datasets were disposed by R 3.6.2 software (https://www.r-project.org/).

### 2.2 Identification of DEIRGs

We matched patients’ clinical information and transcriptomic data according to their ID numbers and removed patients if their ID numbers could not be matched. We ultimately obtained complete gene expression profiles and overall survival (OS) information data from 281 patients. To screen DEIRGs among all identified genes of PRCC, the Wilcoxon signed-rank test was employed to identify differentially expressed mRNAs (DEGs) by the R language limma package based on the cutoff values: *P* value < .05 and |log2 FC| > 1. Then, we screened relative IRGs in DEGs that are DEIRGs.

### 2.3 PDEIRG screening and experimental model construction

Here, the model is first built and trained on the training dataset. The testing dataset means that a dataset was used to provide an unbiased evaluation of a final model fit on the training dataset. The 281 patients were randomly assigned to the training set (n = 139) and the testing set (n = 142). The training dataset was utilized to establish a Cox regression hazards model. Initially, based on the expression profile and OS data, possible PDEIRGs were identified by the R language survival package using univariate Cox analysis. Next, Lasso regression was applied to select the most significant risk genes and eliminate genes that would overfit the model. Finally, we used Cox proportional hazards regression to construct a prognostic risk score model.

### 2.4 Risk score calculation

To calculate the risk score for each patient, we used the regression coefficients from the multivariate Cox regression model to weight the expression values of the selected genes. The following computational formula was used for this analysis:

$$\text{Risk}\;\text{score}=\sum_{\text{i}=1}^{\text{n}}\beta^{\text{i}*}{\text{x}}^{\text{i}}$$

where βi refers to the estimated regression coefficients of each gene and χi represents the expression value of the gene (FPKM). The risk score model was calculated for each patient and used to classify each patient into a low- or high-risk group based on the median risk score of the training dataset as the cutoff. Patients in the low-risk group had a higher OS, and those included in the high-risk group had a lower OS. Kaplan–Meier (KM) survival curves and log-rank tests were used to assess differences in OS between the predicted high- and low-risk groups. The sensitivity and specificity of the diagnostic and prognostic prediction models were analyzed by receiver operating characteristic (ROC) curves and quantified based on the area under the ROC curve (AUC).

### 2.5 Correlation analysis between the risk score and clinical features, immune cell infiltration and tumor mutation burden (TMB)

Univariate and multivariate Cox analyses were performed to test the risk score and clinical features (age, sex, stage) as individual indicators. Then, 3- and 5-year survival rates predicted by the ROC curves for four models were compared.

To explore the associations between the prognostic model and immune cell infiltration, we employed the Cistrome algorithm. Cistrome, a useful resource for comprehensive analysis of tumor infiltrating immune cells, is used for quantifying the composition of six tumor infiltrating immune cell subsets (B cells, CD4+ T cells, CD8+ T cells, neutrophils, macrophages and dendritic cells). The immune infiltrate levels of patients with PPRC were derived from the Cistrome website, and the correlation between the risk score and immune cell infiltration was conducted in R.

Finally, we illustrated the respective mutation profiling of the two risk levels by the R language Maftools package. The TMB in PRCC was defined as TMB = (total count of variants) / (the whole length of exons).

### 2.6 Statistical analysis

All analyses were conducted using R software, and *P* < .05 was considered statistically significant. The rank correlation among the different variables was assessed with the Pearson correlation coefficient test. Differences between variables were assessed with independent *t*-tests. Kaplan-Meier curves and log-rank tests were used to analyze the survival data, and univariate Cox regression analysis was used to identify factors affecting the survival of patients. Multivariate Cox regression analysis was used to identify independent prognostic factors. Time-dependent ROC analysis was used to evaluate the accuracy of the prognostic prediction model. An AUC > 0.60 was regarded as acceptable for predictions, and an AUC > 0.75 was deemed to have excellent predictive value.

## 3 Results

### 3.1 Screening of DEGs and DEIRGs in PRCC

**Figure 1:**
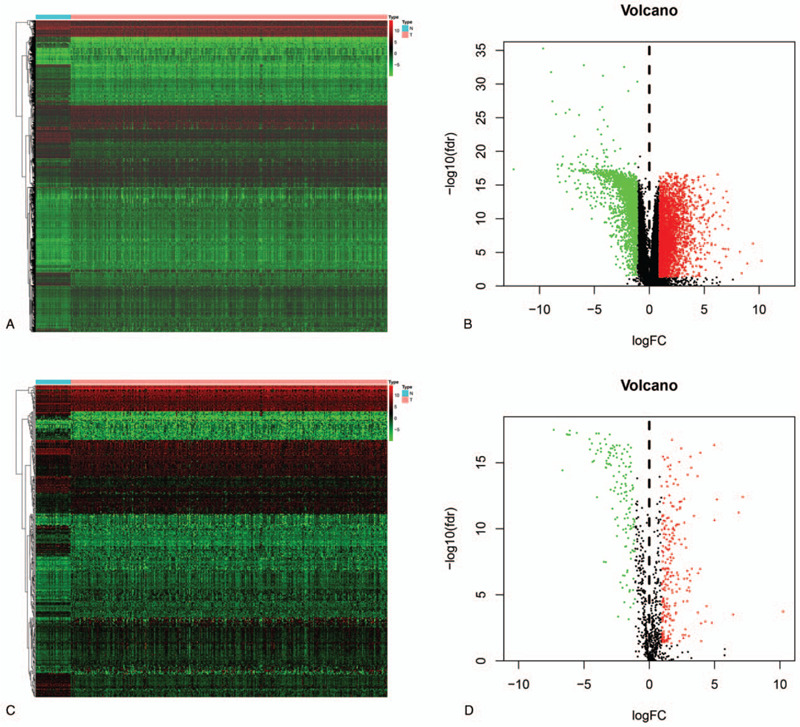
Expression of DEGs and IRGs in the two sample groups. (A and B). Expression of DEGs in the two sample groups represented by a heatmap and Volcano plot. (C and D). Expression of IRGs in the two sample groups represented by a heatmap and Volcano plot. Green represents downregulated genes, black represents non-differentially expressed genes, red represents upregulated genes (*P* value < .05 and |log2 FC| > 1).

The RNA sequencing dataset of patients with PRCC was downloaded from the TCGA database, and the IRGs were obtained from the ImmPort database. A total of 321 samples were used, which included PRCC tissues (n = 289) and normal kidney tissues (n = 32). Using a P value of less than 0.05 and [log FC] more than 1 as the cutoff criteria, we extracted DEGs between PRCC tissues and normal kidney tissues by the R language limma package. A total of 5286 DEGs were identified, including 3370 upregulated and 1916 downregulated DEGs (Fig. 1A and B). Next, IRGs were identified from the extracted DEGs. This analysis revealed 371 differentially expressed IRGs (DEIRGs), including 232 genes that were upregulated and 139 genes that were downregulated in PRCC tissues compared with normal kidney tissues (Fig. 1C and D).

### 3.2 Identification of prognostic DEIRGs and validation of the prognostic gene signature

**Figure 2:**
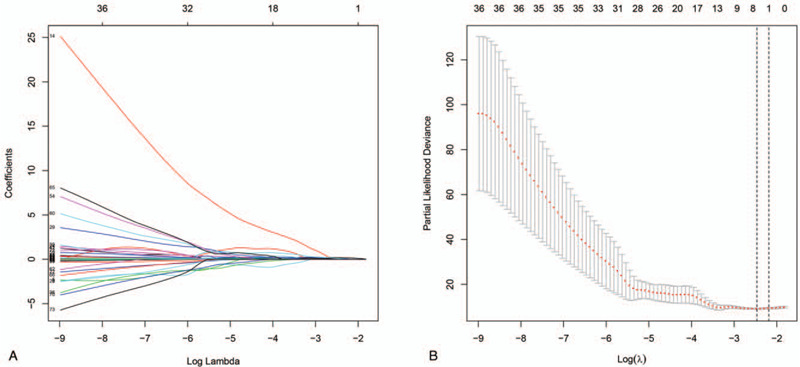
Further analysis of the PDEIRGs in the training cohort. (A and B). Candidate PDEIRGs selected through Lasso regression analysis.

To identify possible prognostic DEIRGs (PDEIRGs), univariate Cox regression analysis was applied to the expression of each DEIRG in the entire TCGA cohort. The results showed that 78 DEIRGs were significantly associated with the overall survival (OS) of patients with PRCC (*P* < .05). We further screened the optimal PDEIRGs to construct a Cox regression hazards model in a training cohort that randomly selected 139 of the 281 patients. First, we used least absolute shrinkage and selection operator (Lasso) regression to delete PDEIRGs that correlated highly with one another and identified 14 candidate PDEIRGs in the training cohort (Fig. 2A and B). The prognostic gene signature was then established based on multivariate Cox proportional hazards regression analysis, and we ultimately defined four optimal PDEIRGs (risk genes) for inclusion in the prognostic risk model: IL13RA2, CCL19, BIRC5, and INHBE. They were identified as high-risk genes (predicting a poor prognosis) in terms of the OS of patients (Table 1).

**Table 1.** Risk genes in the prognostic risk model.

Gene	Coefficient	*P*-value	HR (95% CI)
BIRC5	0.2913	3.30E-06	1.338 (1.184–1.513)
CCL19	0.0157	3.83E-04	1.016 (1.007–1.025)
INHBE	0.0352	.016	1.036 (1.007–1.066)
IL13RA2	0.3711	.036	1.449 (1.025–2.050)

CI = confidence interval, HR = hazard ratio.

### 3.3 Construction of the prognostic risk model in the training set

To investigate the association between the risk genes and the clinical prognosis of patients with PRCC, we developed a prognostic risk scoring system based on these genes. The risk score was calculated as follows:

Risk score=(0.3711*IL13RA2)+(0.0157*CCL19)+(0.2913*, BIRC5)+(0.0352*INHBE).

**Figure 3:**
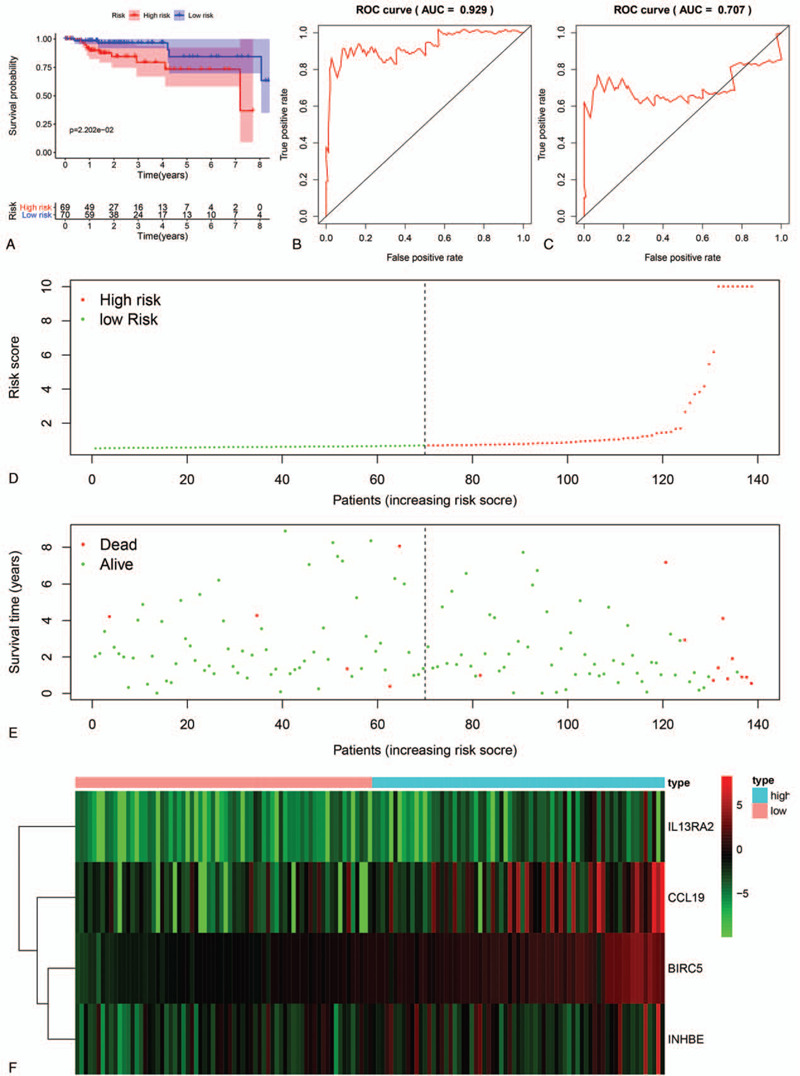
Prognostic analysis of the training set. (A). Survival curve for the low-risk and high-risk groups. (B and C). 3- and 5-year ROC analysis predicted overall survival using the prognostic model. (D). Risk score distribution of patients with PRCC in the prognostic model. (E). Survival status and duration for patients in the prognostic model. (F). Heatmap of the four risk gene expression levels in the prognostic model.

The patients in the training set were then divided into a high-risk group (n = 69) and a low-risk group (n = 70) according to the median risk score. From the Kaplan-Meier curve, patients in the high-risk group had significantly poorer OS outcomes than those in the low-risk group (log-rank test, *P* < .05) (Fig. 3A). The area under the ROC curve (AUC) values for the prognostic model were 0.929 at three years and 0.707 at five years (Fig. 3B and C), confirming the prediction accuracy of survival prediction based on our four-gene signature. The distribution of the risk scores, survival status, and expression of the 4 IRGs in training samples are illustrated in Figure 3D–F. In patients with high-risk scores in the training set, the four risk genes (IL13RA2, CCL19, BIRC5, and INHBE) were upregulated, and these risk genes displayed the opposite expression pattern in the low-risk group.

### 3.4 Validation of the prognostic model in the testing set and the entire TCGA dataset

To assess the performance of the prognostic risk model, we validated our four-gene signature in the testing set (n = 142) and entire TCGA dataset (n = 281). First, the survival risk score of each patient in the testing set and entire TCGA dataset were calculated based on the above formula. According to the median risk score, we divided patients into high- and low-risk groups. In the testing set, 78 patients were categorized as high-risk and 64 were categorized as low-risk. In the entire TCGA dataset, 145 patients were classified as high-risk and 136 were classified as low-risk.

**Figure 4:**
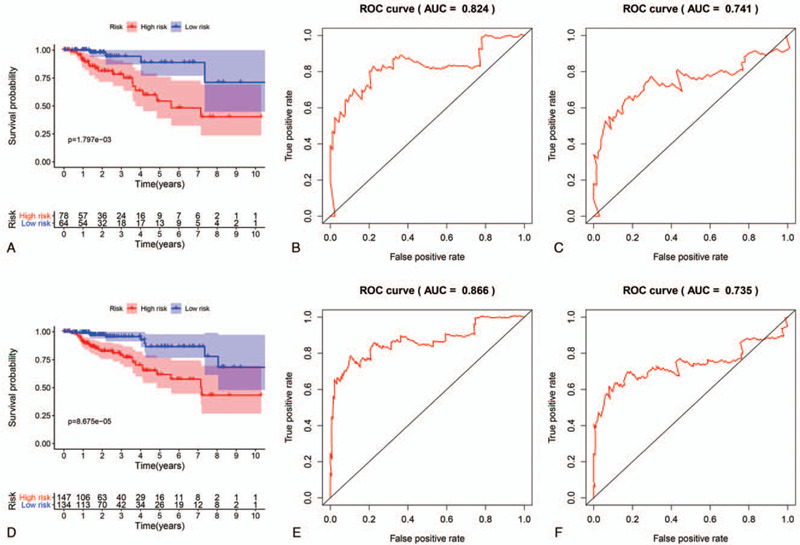
Validation of the prognostic model in the testing set and the entire TCGA dataset. (A). Kaplan-Meier curve analysis of high-risk and low-risk patients in the testing set. (B and C). Time-dependent ROC curve for predicting the 3- and 5-year survival in the testing set. (D). Kaplan-Meier curve analysis of high-risk and low-risk patients in the entire TCGA set. (E and F). Time-dependent ROC curve for predicting the 3- and 5-year survival in the entire TCGA set.

Next, Kaplan-Meier survival analysis was used to determine the prognostic differences between the high-risk and low-risk groups. The Kaplan-Meier curve results showed that there was a significant difference in prognosis between the two groups in both the testing set and the entire TCGA dataset (*P* < .05) (Fig. 4A and D). In agreement with the results of the training set, the survival curves demonstrated that patients in the high-risk group exhibited markedly poorer overall survival than those in the low-risk group. Moreover, time-dependent ROC analyses were performed for the testing set and the entire TCGA dataset at three and five years. In the testing set, the AUCs at three and five years were 0.824 and 0.741, respectively (Fig. 4B and C). In the entire TCGA set, the AUCs at three and five years were 0.866 and 0.735, respectively (Fig. 4E and F).

**Figure 5:**
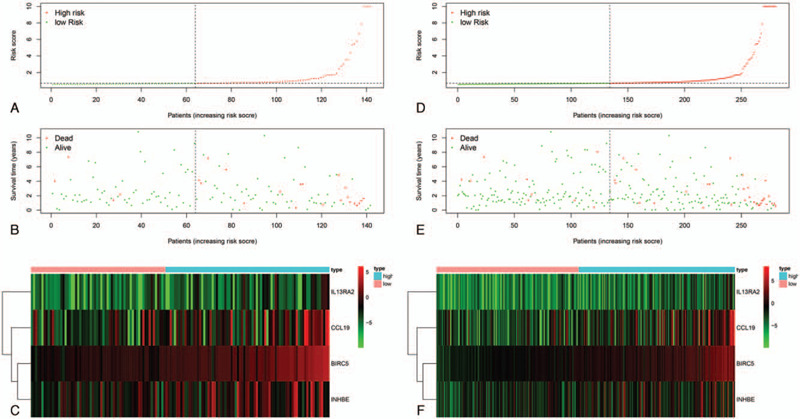
Prognostic analyses of high-risk and low-risk patients in the testing set and the entire TCGA dataset. (A). Risk score distribution of patients in the testing set. (B). Survival status scatter plots of patients in the testing set. (C). Heatmap of risk gene expression in the testing set. (D). Risk score distribution of patients in the entire TCGA set. (E). Survival status scatter plots of patients in the entire TCGA set. (F). Heatmap of risk gene expression in the entire TCGA set.

The risk score distribution, survival status and risk gene expression in the testing set and the entire TCGA dataset are displayed in Fig. 5A–F. Similar to the results in the training set, the four-gene levels were lower in the low-risk group than in the high-risk group. Therefore, these results demonstrated that this four-gene risk model can be used and is precise regarding the prognostic prediction of patients with PRCC.

### 3.5 Independence of the risk model from other clinical factors

**Figure 6:**
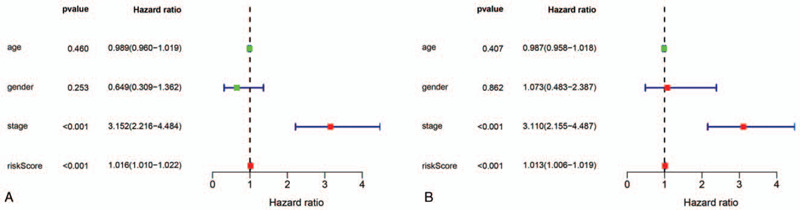
Forest plot summary of analyses of OS. (A). Univariate analysis of the risk score, age, sex and pathological stage in the TCGA dataset. (B). Multivariate Cox analysis of the TCGA dataset.

We speculate that the collaboratively abnormal expression of the risk model could be regarded as an independent prognostic factor. Next, univariate and multivariate Cox regression analyses were conducted to assess whether the risk score model was independent from other clinical parameters (age, sex and pathological stage) as a prognostic factor for PRCC. The univariate analysis indicated that pathological stage and our risk score model (*P* < .001) were markedly correlated with the prognosis of patients with PRCC (Fig. 6A). The multivariate analysis revealed that the risk score and stage remained independent prognostic factors associated with OS in the entire TCGA dataset (*P* < .001, Fig. 6B and Table 2). These results demonstrated that the prognostic risk model and pathological stage can be used independently to predict the prognosis of patients with PRCC.

**Table 2.** Univariate and multivariate Cox regression analyses of the entire TCGA cohort.

	Univariate analysis		Multivariate analysis	
Variables	HR (95% CI)	*P*-value	HR (95% CI)	*P*-value
	Overall survival			
Age	0.99 (0.96–1.02)	.460	0.99 (0.96–1.02)	.407
Gender	0.65 (0.31–1.36)	.253	1.07 (0.48–2.39)	.862
Stage	3.15 (2.22–4.48)	1.69E-10	3.11 (2.16–4.49)	1.32E-09
Risk score	1.02 (1.01–1.02)	9.90E-07	1.01 (1.01–1.02)	2.23E-04

CI = confidence interval, HR = hazard ratio, TCGA = The Cancer Genome Atlas.

**Figure 7:**
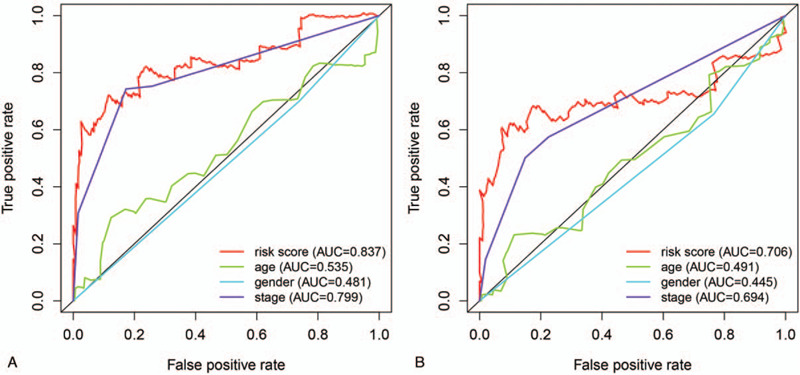
Time-dependent ROC curve analyses of risk score and clinical factors in the entire TCGA dataset at three and five years. (A). AUC at three years. (B). AUC at five years.

We also performed 3- and 5-year ROC analyses to determine the sensitivity and specificity of these factors. The risk score was more accurate than the other clinical parameters: the AUCs at three years for risk score, age, sex and pathological stage were 0.837, 0.535, 0.481 and 0.799, respectively (Fig. 7A), and the corresponding variables at five years were 0.706, 0.491, 0.445 and 0.694, respectively (Fig. 7B).

### 3.6 Correlation among immune cell infiltration, TMB and the risk score

**Figure 8:**
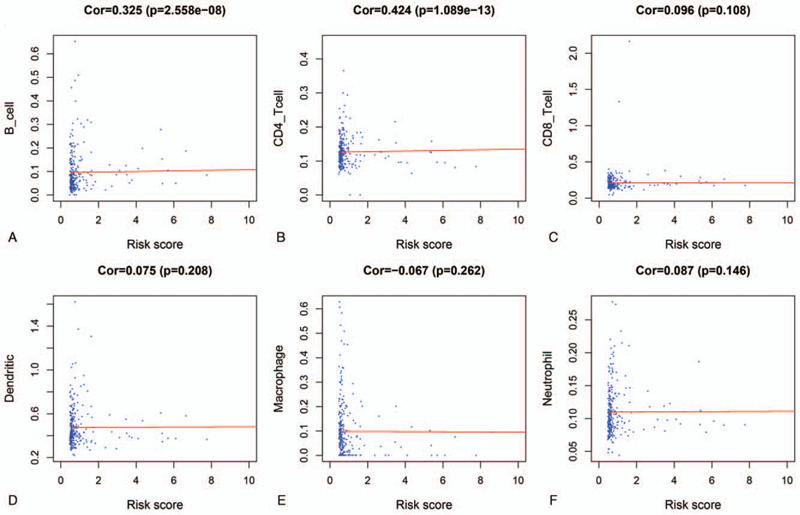
Relationships between the risk score and infiltration abundances of six types of immune cells. (A). B cells. (B). CD4+ T cells. (C). CD8+ T cells. (D). Dendritic cells. (E). Macrophages. (F). Neutrophils.

**Figure 9:**
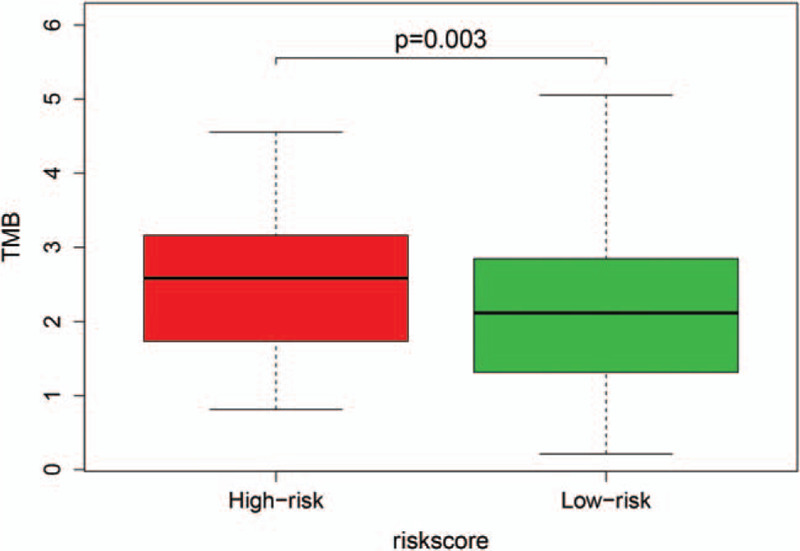
Mutation burden of patients with PRCC in the high-risk and low-risk groups with risk score.

To determine whether our risk model could reflect the tumor immune microenvironment and mutation burden in patients with PRCC, immune infiltration profiling and mutation count analyses were carried out to explore the correlation between the risk score and immune cell infiltration and tumor mutation burden (TMB) in the entire TCGA dataset. We found that the abundance of B cells and CD4+ T cells was positively correlated with the risk score (*P* < .05, Fig. 8A and B). However, no significant correlations were observed between CD8+ T cells, dendritic cells, macrophages, neutrophil cells and risk score (Fig. 8C–F). Compared with the low-risk group, the mutation count was higher in the high-risk group of patients with PRCC (*P* < .05, Fig. 9).

## 4 Discussion

Because of the different molecular mechanisms and the low proportion of PRCC in RCC, patients with PRCC have been excluded from large clinical trials of targeted drugs, such as sorafenib and sunitinib, and research on PRCC is always less studied than clear cell RCC and progresses slowly.^[21]^ Although some patients with PRCC can be diagnosed by ultrasonography and receive surgery at an early stage, a significant number of advanced patients die due to postoperative recurrence, metastasis, and resistance to chemotherapeutic drugs, underscoring the importance of exploring the molecular mechanisms and prognostic factors of PRCC.^[22]^

Recently, studies have been reported involving gene signatures for prognostic prediction in human cancers.^[23–25]^ Immune cells were found in human solid tumors, and the immune pattern of the tumor microenvironment is a major predictor of patient survival in most primary tumors.^[26]^ Previous studies have shown a major prognostic value of the immune pattern (CD8+/DC-LAMP+ cell densities) in colorectal carcinoma and RCC, reproducible from primary to metastatic tumors.^[27]^ The immune checkpoint molecules on expanded T cells in patients with advanced RCC were higher than those on unexpanded T cells before surgery.^[28]^ Considering the importance of the immune environment in the progression of cancer, it is essential to identify immune-related biomarkers to evaluate the prognosis of patients with PRCC, which may also play a significant role in immunotherapy. The objective of our study was to recognize the immune-related genes (IRGs) associated with prognosis and to construct a dependable model to predict the overall survival (OS) of patients with PRCC.

First, we obtained 371 DEIRGs, including 232 upregulated and 139 downregulated genes, based on 289 PRCC tissues and 32 normal kidney tissues that were downloaded from the TCGA database. We then performed Cox and Lasso regression analyses to assess the relationship of these DEIRGs with the prognosis of patients with PRCC, and 4 PDEIRGs of interest (IL13RA2, CCL19, BIRC5, and INHBE) were ultimately determined. All 4 PDEIRGs have been reported to be involved in the immune microenvironment and inflammatory response.^[29–32]^ The IL-13RA2 bound and upregulated by IL-13 as well as CCL19 ligand binding the chemokine receptor CCR7, they have been established as an important component of migratory events in adaptive immune function, such as intravenous transfer of CD4 and CD8 T cells.^[33,34]^ Kuo et al showed anti-apoptotic protein BIRC5 maintained survival of HIV infected CD4 T cells and Li et al found that BIRC5 and INHBE were significantly overexpressed in the high TMB group and correlated with worse prognosis in chromogenic RCC (chRCC).^[35,36]^ Moreover, IL13RA2 could mediate resistance to sunitinib in certain populations of ccRCC by avoiding sunitinib-induced apoptosis.^[37]^ BIRC5 was overexpressed in patients with breast cancer and was responsible for shorter relapse-free survival, worse overall survival, reduced distant metastasis-free survival, and increased risk of metastatic relapse events.^[38]^ In addition, INHBE emerged as a candidate hepatokine associated unexpectedly with whole-body energy metabolism under obese insulin-resistant conditions, which could decrease fat utilization and increase fat mass.^[39]^ CCL19 has been regarded as one of the immune-related risk genes that can predict PRCC patient survival.^[18]^ From this, a prognostic prediction model was constructed, and the risk score of patients was calculated.

Next, we examined the value of the risk score model for the prognostic prediction of patients by survival analysis. The results showed that patients in the high-risk group had significantly poorer OS outcomes than those in the low-risk group, suggesting that the model was associated with the prognosis of patients with PRCC. We then further analyzed the reliability and stability of the model and validated it. Our results indicated that the model could accurately discriminate patients with different survival outcomes. Combining univariate and multivariate Cox regression analyses, the model was demonstrated to independently predict the prognosis of patients with PRCC. Thus, our model can be used to identify patients with PRCC at high risk for death and to carry out early interventions to improve the prognosis of patients in clinical work.

Previous studies have demonstrated that immune infiltration is an important determinant of the therapeutic response and prognosis of cancer.^[40,41]^ Li et al found that higher enrichment of multiple immune/inflammatory cells, such as Th2 cells and macrophages, was associated with poor prognosis in breast cancer.^[42]^ G. Drake et al reported that the high infiltration of CD8+ T cells in RCC is related to worse outcome.^[43]^ Therefore, we also analyzed the relationship between the risk score and immune cell infiltration and found that the risk score correlated positively with the infiltration of B cells and CD4+ T cells. The tumor mutation burden (TMB) might predict clinical response and be associated with survival in patients taking immune checkpoint inhibitors (ICIs) across a wide variety of cancer types.^[44,45]^ Thus, we speculated whether our model reflected TMB and found that the mutation burden was higher in the high-risk group than in the low-risk group. These results suggested that the model can be used to distinguish patients with different sensitivities to immunotherapy and to develop individualized treatment strategies.

Recently, the risk models according to PDEIRGs have attracted wide attention and revealed the tremendous potential in prognosis prediction of patients with cancer. Wang et al constructed a prognostic risk model screening 15 PDEIRGs in PRCC and verified that the model could independently distinguish patients with different risks of death.^[18]^ Wan et al applied Cox and Lasso regression to identify 7 PDEIRGs for establishing a risk model for the prognostic stratification of patients with ccRCC and found that the model could predict immune cell infiltration, the mutation burden and the progression of ccRCC.^[17]^ Zhang et al established a prognostic prediction risk score model based on the expression profiles of 14 IRGs in lung adenocarcinoma that showed high prediction accuracy and stability in identifying immune features.^[46]^ Our research differed from previous studies in several ways. First, there were fewer IRGs in our model than in the previous models, and we focused on only 4 IRG expression patterns in PRCC. Second, the IRGs in our model did not overlap with those in the previous models. Third, we used multiple algorithms (including univariate Cox, multivariate Cox and Lasso regression) to identify PDEIRGs for the model. Therefore, our study was more accurate and reliable than the others.

There are still some limitations in our study. First, all of the investigative data were completely acquired from public databases. Second, we evaluated the performance of the risk model by the full TCGA cohort lacking further validation, owing to limited patient numbers in the validation datasets. Third, the biological functions of 4 PDEIRGs in PRCC require further examination by a series of experiments.

## 5 Conclusion

In summary, we constructed a risk model using 4 IRGs (IL13RA2, CCL19, BIRC5, and INHBE) for the prognostic prediction of patients with PRCC from the TCGA database. The risk score generated by this model can serve as an independent prognostic predictor to distinguish patients with different survival outcomes for PRCC. Moreover, this prognostic model may also serve as a predictor for increased immune cell infiltration (B cells and CD4+ T cells) and can stratify patients with PRCC with different mutation burdens. Our study develops knowledge of IRGs in PRCC and provides new potential prognostic and therapeutic biomarkers. However, further experiments are required to verify the findings of this study.

## Author contributions

**Conceptualization:** Leilei Wang, Huijun Ni.

**Data curation:** Leilei Wang, Weile Gu, Huijun Ni.

**Formal analysis:** Leilei Wang, Weile Gu, Huijun Ni.

**Project administration:** Huijun Ni.

**Resources:** Leilei Wang.

**Software:** Leilei Wang.

**Supervision:** Weile Gu, Huijun Ni.

**Validation:** Weile Gu, Huijun Ni.

**Visualization:** Leilei Wang, Weile Gu, Huijun Ni.

**Writing – original draft:** Leilei Wang.

**Writing – review & editing:** Weile Gu, Huijun Ni.
